# The Effects of CenteringPregnancy: A Quasi-Experimental Evaluation

**DOI:** 10.3390/healthcare13091052

**Published:** 2025-05-03

**Authors:** Chul Hyun Park, Nichola Driver, Robert C. Richards, Penny Ward

**Affiliations:** 1 School of Public Policy, University of Maryland, Baltimore County, 1000 Hilltop Circle, Baltimore, MD 21250, USA; 2 Clinton School of Public Service, University of Arkansas, 1200 President Clinton Avenue, Little Rock, AR 72201, USA; nddriver@clintonschool.uasys.edu (N.D.); rcrichards@clintonschool.uasys.edu (R.C.R.J.); 3 Psychiatric Research Institute, University of Arkansas for Medical Sciences, 4301 W Markham St, Little Rock, AR 72205, USA; pward@uams.edu

**Keywords:** CenteringPregnancy, group prenatal care, quasi-experimental design

## Abstract

**Background/Objectives**: CenteringPregnancy (CP) is a group prenatal care model that empowers pregnant women through risk assessment, education, and social support. CP participants have more contact time with providers compared to those in traditional prenatal care. Despite contradictory findings in the literature, this study aimed to determine if CP leads to better health outcomes for women and their infants. **Methods**: A quasi-experimental design was used. Data were collected from hospital birth records of both CP participants and those receiving traditional prenatal care from 2018 to 2020. Various treatment effect models for observational data were used to assess CP’s effectiveness. **Results**: CP improved women’s access to prenatal care and reduced rates of preterm birth and perinatal death compared to traditional prenatal care. It also resulted in longer gestation periods and better infant birth weight outcomes. However, no significant differences were found in delivery type (vaginal or C-section) or breastfeeding initiation and continuation between the two groups. **Conclusions**: These findings provide compelling evidence that CenteringPregnancy can lead to significantly improved maternal and infant health outcomes by demonstrating its effectiveness in a real-world clinical setting.

## 1. Introduction

The CenteringPregnancy (CP) model of prenatal care is designed to improve health outcomes for disadvantaged women and their babies. The model diverges from traditional forms of prenatal care by bringing pregnant women together for group prenatal care sessions. Developed by a Certified Nurse Midwife, Sharon Schindler Rising, the program incorporates the three standard components of prenatal care—“risk assessment, education, and support”—into the group setting [1] (p. 46). Pregnant women are placed into groups of 8–12 women with similar due dates and participate in 10 sessions throughout their pregnancy, each lasting 90–120 min, a substantial increase compared to the traditional model, where they receive 10–15 min [1]. Upon arrival, women participate in self-assessments by measuring their own weight and blood pressure; then, the prenatal exam is conducted by a clinician (nurse-midwife, nurse practitioner, or physician). During each individual exam, the remaining women have the chance to chat, review their charts, and fill out worksheets related to the upcoming discussion. The remaining time is spent on group discussion, which includes topics such as “nutrition, early pregnancy concerns and self-care, substance abuse, preparation for childbirth, adaptation to the postpartum period, infant feeding, contraception, and parenting” [2] (p. 407).

The program integrates the three standard components of prenatal care—prenatal checkups, health education, and facilitative discussion—into a group setting [3]. The model is designed to encourage peer support and free exchange among participants and facilitate increased learning about health during pregnancy.

Over the past two decades, numerous studies have been conducted to determine whether the CP model yields positive health outcomes for mothers and their infants [4,5,6]. However, these studies have reported mixed results, regardless of the research design employed (experimental or quasi-experimental) [7]. While some studies have found empirical evidence supporting the effects of CP on certain health outcomes [8], others have not found statistically significant evidence to support these findings [9]. These discrepancies may stem from shortcomings in previous research, including notably small sample sizes, inadequate controls, and the absence of sensitivity analyses. Consequently, there is a clear need for more rigorous empirical research to examine the effects of CP on various health outcomes for mothers and their babies.

To address the limitations of earlier investigations, this study employs large sample sizes, includes covariates to control for differences between CP participants and non-participants, and utilizes multiple analytical methods to answer the following research question: does the CenteringPregnancy model, compared to traditional prenatal care, yield significantly better outcomes for women and their infants in the areas of prenatal visit attendance, gestational age, preterm birth, delivery type, infant birth weight, gestational weight gain, perinatal death, and breastfeeding initiation and continuation?

## 2. Materials and Methods

This study employs a quasi-experimental evaluation. Using de-identified hospital birth records, maternal and infant outcomes were extracted for both CP participants (n = 216) and traditional prenatal care/non-CP participants (n = 1159) from a hospital-affiliated public clinic in Little Rock, Arkansas. The clinic started offering CP in August 2018. Pregnant patients were recruited to the CP program when scheduling their initial prenatal appointments. They were offered a choice of traditional prenatal care or CP. 

The current study included women aged 17 and older who delivered an infant at the health clinic between August 2018 and March 2020, who entered prenatal care at 24 weeks or less, and whose pregnancies were not considered “high risk” (such as those with diabetes or HIV). Data were provided to the evaluators as de-identified hospital records that were recorded by clinic staff. Methods for this study were approved by the Institutional Review Boards at the University of Arkansas for Medical Sciences and the University of Maryland, Baltimore County.

### 2.1. Measures

The primary health outcomes of interest include attendance at prenatal care visits, gestational age, preterm birth, perinatal death, delivery type, infant birth weight, gestational weight gain, and breastfeeding initiation and continuation. Prenatal visit attendance was measured as the total number of prenatal care visits attended [10]. Gestational age was measured in weeks, determined by the last menstrual period [11]. Preterm birth was a dichotomous variable (1 = less than 37 weeks) [12]. Perinatal death refers to fetal or neonatal demise (1 = demise) [13]. Delivery type was a dichotomous variable (1 = vaginal delivery and 0 = cesarean section) [13]. Infant birth weight was measured in ounces [4]. Two dichotomous variables were created for infant birth weight measures [10]: low birth weight (1 = less than 88.18 ounces) and very low birth weight (1 = less than 52.91 ounces). Gestational weight gain was a continuous variable measured in pounds [13]. Breastfeeding initiation and continuation are both dichotomized variables (1 = exclusive breastfeeding at discharge and 1 = exclusive breastfeeding at 6-week postpartum appointment) [14].

Other variables included in the study were the mother’s race, ethnicity, age, BMI, and insurance type (a proxy measure for socioeconomic status). These variables served as covariates or matching variables. Race was dichotomized as 1 = Black and 0 = non-Black. Ethnicity was also dichotomized as 1 = Hispanic/Latina and 0 = non-Hispanic/Latina. Age was a continuous variable. BMI was dichotomized as 1 = overweight or obese and 0 = underweight or normal weight, using a BMI cut-off point of 25. Insurance type was dichotomized as 1 = private insurance and 0 = public insurance.

### 2.2. Analytical Methods

To reduce selection bias in estimating the average treatment effects (ATEs) [15], this study employed robust analytical methods. Although quasi-experimental evaluation is likely to be vulnerable to selection bias, many prior quasi-experimental evaluations on CP did not include covariates for their analytical models. In addition, most previous studies employed only one type of analytical model. Thus, five different treatment effect models for observational data were employed to minimize selection bias: regression adjustment (RA) [15]; augmented inverse-probability weights (AIPWs) [16]; inverse-probability-weighted regression adjustment (IPWRA) [15]; nearest-neighbor matching (NNM) [17]; and propensity score matching (PSM) [18]. This approach allowed the study to triangulate treatment effect estimates and assess whether the results remained consistent or diverged across models, serving as a robustness check through methodological triangulation. It also enabled a sensitivity analysis to evaluate how the treatment effect estimates responded to different model assumptions and estimation techniques.

RA uses a regression model to estimate the difference between potential outcomes for the treatment group and the comparison group. Specifically, for continuous outcome variables, linear regression (gestational age, infant birth weight, and gestational weight gain) or Poisson regression (prenatal visits) was used. Logistic regression was used for binary outcomes (preterm birth, perinatal death, delivery type, low birth weight, very low birth weight, and breastfeeding initiation and continuation). AIPW and IPWRA are called “doubly robust” methods because they are based on a combination approach that uses simultaneously RA and inverse-probability-weights correcting for a missing data problem [15]. Treatment effects were estimated using two one-to-one matching methods based on nearest neighbors and the propensity score. This study considered age, dichotomized BMI, race, ethnicity, and insurance type as covariates or matching variables. Regarding statistical software, Stata 17 was used for all models.

## 3. Results

Table 1 shows summary statistics on covariates or matching variables as well as key outcome variables. Regarding dichotomized BMI, there was no statistically significant difference between the CP group and the traditional prenatal care group. However, concerning race, there were more Black women in the CP group compared to the traditional prenatal care group (*p* < 0.001). In addition, the two groups were different regarding ethnicity, age, marital status, and insurance type (*p* < 0.01). Therefore, those variables were included in the treatment effect estimation models as covariates or matching variables to control for potential confounding factors.

Table 2 represents results estimated by different treatment effect models. Importantly, there are statistically significant beneficial effects of CP on the following health outcomes: prenatal care visits, gestational age, preterm birth, perinatal death, infant birth weight, low birth weight, and very low birth weight. Furthermore, all of these same outcomes remained statistically significant across all treatment effect models. For the purpose of interpretation, the most conservative estimate for each outcome variable—i.e., the estimated coefficient with the smallest absolute value—is presented below.

Participation in CP significantly increased the number of prenatal care visits attended by approximately 1.85 (propensity score matching model, *p* < 0.001). Also, participation in CP is associated with a 1.23-week increase in gestational age (37.99 weeks for the CP group vs. 36.76 weeks for the traditional care group, regression adjustment model, *p* < 0.001). Additionally, on average, participation in CP reduced the rates of preterm birth by 11.9 percentage points (regression adjustment model, *p* < 0.001). Furthermore, involvement in CP led to an average reduction of approximately 3.6 percentage points in the perinatal death rate (nearest-neighbor matching model, *p* = 0.002).

Next, the average infant birth weight is approximately 10.8 ounces more if all the women had participated in CP compared to the average of 101.9 ounces that would have occurred if these women had used traditional prenatal care (regression adjustment model, *p* < 0.001). Second, participation in CP is associated with an average reduction of 12 percentage points in the probability of having low-birth-weight babies (less than 88.18 ounces) (regression adjustment model, *p* < 0.001). Additionally, the likelihood of having babies with a very low birth weight (less than 52.91 ounces) decreases by 6.4 percentage points when participating in CP compared to the probability with traditional prenatal care (regression adjustment model, *p* < 0.001).

Nonetheless, concerning delivery type (vaginal delivery vs. cesarean section), there was no significant difference between women who attended CP and those in traditional care across all the treatment effect models. Furthermore, with respect to both breastfeeding initiation at discharge and breastfeeding continuation at 6 weeks postpartum, the study did not find statistically significant evidence to support the effects of CP. Finally, this study found that women who participated in CP gained significantly more weight (3.17 pounds) compared to those in traditional care (propensity score matching model, *p* = 0.037).

## 4. Discussion

This section will describe the study’s rationale and main findings, the unique aspects of the study, the study’s primary significance and contributions, and the study’s subjects. Next, findings from previous studies will be compared with those of this study for each of the outcome variables examined. Finally, the study’s limitations will be described and clinical implications explored.

Prior empirical studies of CP have yielded mixed results for most outcome variables concerning the health of postpartum women and infants. Those inconsistent results may arise from weaknesses in those studies’ methodologies, particularly concerning inadequate sample sizes, a lack of control variables, and a failure to test the robustness of results. This study sought to remedy those methodological flaws by investigating the influence of CP on maternal and neonatal health outcomes with a more rigorous methodology. This inquiry addressed the following research question: does the CP model of group prenatal care, compared to traditional individualized prenatal care, result in significant improvements in maternal and neonatal health with respect attendance at prenatal appointments, gestational age, preterm birth, delivery type, infant birth weight, gestational weight gain, perinatal death, and breastfeeding initiation and continuation?

The results of this study showed that CP had significant, positive effects on prenatal care visits, gestational age, and infant birth weight, as well as significant, negative impacts on preterm birth, perinatal death, and low and very low birth weight. However, CP had no significant effect on the rate of cesarean section or breastfeeding initiation or continuation. Additionally, these findings revealed a significant, positive association between CP and gestational weight gain.

The uniqueness of this study lies in its methodological rigor. In some prior observational studies, analyses of the effects of CP on outcomes were undertaken without controlling for some significant differences between intervention and control groups [19,20]. Furthermore, some studies may have been underpowered due to relatively small sample sizes [20,21,22]. Additionally, in only a few previous studies did researchers perform sensitivity testing using a variety of alternative statistical techniques [5,23,24]. To address those shortcomings, this study sought to include covariates in the analyses of relationships between groups and outcomes to control for significant between-group differences, relatively large sample sizes to enable the detection of even small effects, and up to five different analytical techniques to test the robustness of the results [15,16,17,18].

Prior literature presents conflicting findings regarding the effects of participation in CP on gestational age, preterm birth, delivery type, infant birth weight outcomes, and perinatal death.

Specifically, with respect to gestational age, several observational studies found that participation in CP was associated with longer gestation, potentially due to higher levels of social support experienced within CP groups [11,23,25,26]. Conversely, many other studies reported no significant impact of CP on gestational age [9,12,13,20,27,28,29]. Additionally, several randomized controlled trials (RCTs) and observational studies found that mothers assigned to CP were less likely to give birth to premature neonates compared to mothers in traditional care [12,25,26,29,30]. However, many other studies failed to find any significant evidence to support the effects of CP on reducing preterm birth [6,9,11,13,19,21,29,31].

Further, several prior studies discovered that there was a significant difference in cesarean section rates between CP participants and non-participants [4,13,19,32]. Contrary to this finding, other studies found no significant evidence that CP reduced the incidence of cesarean delivery [6,9,21,28,29,30]. With respect to infant birth weight, a number of previous studies revealed a significant, positive effect of CP on infant birth weight and a significant reduction in the rate of low birth weight infants [4,11,13,23,30]. Yet, some studies have reached the opposite conclusion [6,8,9,12,19,25,26,31]. Moreover, a number of earlier investigations indicated that CP participation had no significant effect on perinatal death [4,11,12,13], yet one study found that CP significantly reduced fetal death [23].

Among those five outcome variables, the current study identified significant evidence supporting the effects of CP on gestational age, preterm birth, infant birth weight-related outcomes, and perinatal death. These findings, produced by means of a more rigorous methodology than had been used in most earlier research, contribute to the literature by providing stronger empirical evidence of the association between CP and health outcomes.

The subjects of this study were women aged 17 years or above who gave birth at a clinic in Little Rock, Arkansas, USA, from August 2018 through March 2020, began prenatal care before the 24th week of pregnancy, and who did not have conditions, such as HIV or diabetes, that rendered their pregnancies “high risk”. Members of the treatment group participated in CP group prenatal care (n = 216), whereas members of the control group received traditional, individualized prenatal care (n = 1159).

With respect to countries neighboring the U.S., in a study conducted in Canada, Benediktsson and colleagues found no significant effect of CP on breastfeeding initiation or continuation [33], consistent with the findings of this study. In the Netherlands—another high-income country where CP research has been conducted—Wagijo and colleagues’ research yielded two results that accord with findings reported here: that CP significantly increased prenatal care visits [34] but had no significant effect on the rate of cesarean delivery [6]. Nonetheless, Wagijo and colleagues produced other findings that conflicted with this study’s results, namely that CP significantly increased breastfeeding initiation while having no significant impact on infant birth weight or preterm birth [6].

Regarding comparisons with earlier research, generally, the results of this study align with previous findings that participation in CP increases attendance at prenatal care visits [4,8,19,21,27,30,34,35,36]. Older women’s mentoring of younger women may be a factor, as Earnshaw and colleagues identified a positive association between age diversity within CP groups and the number of prenatal visits, with patient engagement serving as a mediator [10].

In terms of the impact of CP on gestational age, although some previous research detected no such impact [9,12,13,20,27,28,29], this study found that CP had a significant, positive effect on gestational age, consistent with prior observational studies showing that participation in CP was associated with longer gestation, potentially due to higher levels of social support within CP groups [12,23,25,26]. Regarding birth weight, a number of previous studies found that CP had no significant impact on infant birth weight [6,8,9,12,19,25,26,31]. Nonetheless, this study revealed that CP had a significant, positive effect on mean birth weight and led to significant reductions in the rate of low and very low infant birth weight, consistent with several earlier studies [4,11,13,23,30].

Regarding preterm birth, whereas some studies failed to find any significant evidence to support the effects of CP on reducing premature birth [6,9,13,31], this study showed that CP significantly lowered the rate of preterm birth, in accord with several earlier investigations [12,24,25,30]. Further, although some prior studies discovered no significant evidence that CP reduced perinatal death [12,13], Tanner-Smith and colleagues found that women participating in CP had a lower likelihood of fetal death compared to those receiving traditional care [23]. Confirming Tanner-Smith and colleagues’ findings, this study’s results showed that CP participants had a significantly lower rate of perinatal death compared to non-participants. Thus, several results of this study align with prior research demonstrating the beneficial effects of participation in CP on maternal and neonatal health outcomes.

With respect to delivery type, although some previous investigations showed a significant association between CP and cesarean delivery [19,32], most earlier studies found no such link [6,9,21,29], a result confirmed by this study. Moreover, whereas some previous research showed that CP increases breastfeeding initiation and continuation [37,38], other prior studies have revealed no significant impact of CP participation on breastfeeding [33,39,40], a finding corroborated by this study. This lack of effect is unsurprising, since CP primarily focuses on prenatal support rather than postpartum care. Breastfeeding initiation and continuation are complex outcomes influenced by a variety of social and environmental factors external to CP, such as parental leave policies, workplace breastfeeding support, and societal norms regarding breastfeeding [41].

Finally, in terms of gestational weight gain, some impact studies have found that CP participation has a significant, negative effect on gestational weight gain compared to non-participation [19,24]. Nonetheless, most earlier investigations have discovered either no significant effect or, as this study found, that participation in CP is associated with significantly greater gestational weight gain than traditional prenatal care [22,42,43]. The latter finding aligns with results of a recent meta-analysis, which noted that “excessive gestational weight gain was higher in group care compared to traditional care” in the highest-quality studies [44] (p. 11), possibly due to the higher proportion of nulliparous and minority women in group care settings. Further research is needed to explore the underlying mechanisms contributing to greater gestational weight gain in group prenatal care, as well as to identify strategies for ensuring adherence to recommended weight gain guidelines.

In sum, this study demonstrated that participation in CP significantly improved the number of patients’ prenatal care visits, as well as gestational age and infant birth weight, while significantly decreasing the rate of preterm birth, perinatal death, and low and very low birth weight. CP was found to have no significant impact on delivery type or breastfeeding initiation or continuation, however. Moreover, CP was significantly and positively associated with gestational weight gain. Notably, this study yielded clear evidence of the positive effect of CP on four neonatal health outcomes—gestational age, preterm birth, infant birth weight outcomes, and perinatal death—about which previous research had generated mixed results.

The strength of this study lies in the rigor of its methodology, which features substantial sample sizes, covariates to control for differences between control and treatment groups, and the use of five different statistical methods to enable sensitivity analysis [15,16,17,18]. 

Of course, the findings in this study should be interpreted within the context of the study’s limitations. Although this study sought to control for confounding factors that may influence causal inference by utilizing several advanced analytical methods, this study used observational data, and women were not randomly assigned to CP or traditional care. Also, the specific content of CP sessions and the experience or effectiveness of the group prenatal care facilitator could not be evaluated.

Importantly, this study mainly sought to answer whether CP significantly produced expected health outcomes by employing a quasi-experimental design. However, this study was not able to provide sufficient knowledge about how and why CP worked or did not. Most previous studies of CP have not been informed by theory, and little is known about the mechanisms by which CP contributes to health [14]. Therefore, future research needs to explore the factors contributing to program effectiveness, such as social support and stress reduction [45,46,47], mentoring by older mothers [10], social influence [48], cultural and ethnic contexts [49], and the level of engagement by health professionals [50].

The U.S. has poorer maternal care outcomes than most other peer countries, with higher maternal mortality rates, higher infant mortality rates, and the lowest supply of maternal care providers, and U.S. mothers are the least likely to have access to provider home visits or paid parental leave in the postpartum period [51]. Findings that demonstrate the consistent effectiveness of CP can have significant policy implications for maternal and child health, especially among low-income or disadvantaged communities.

Given U.S. maternal disparities, the findings in this study demonstrate a need for policymakers and clinicians to urgently consider prioritizing group prenatal care initiatives and incorporating CP programs across public healthcare facilities, such as community health centers and federally qualified health centers. Furthermore, policymakers should consider expanding insurance coverage for these programs, including Medicaid, in order to increase accessibility and affordability for at-risk populations, thereby addressing disparities in prenatal care [52]. Finally, in addition to patient outcomes, clinicians should consider the impact of CP on their workflows and staffing issues. CP was designed to foster a collaborative approach to obstetric care, enabling providers to see more patients, achieve better prenatal outcomes, and increase availability within clinics [53]. 

## 5. Conclusions

For a generation, the CenteringPregnancy (CP) method of group prenatal care has been implemented with the aim of improving health outcomes for women and infants, especially those from under-resourced and marginalized communities. Yet, doubts have persisted about CP’s effectiveness due to the mixed results of earlier studies. Employing a rigorous methodology, this study furnishes clear evidence that CP, compared to traditional individualized prenatal care, improves several indicators of maternal and infant health. Researchers now have an opportunity to investigate the mechanisms by which CP enhances the well-being of women and neonates, while clinicians and policymakers can act to expand access to this promising mode of prenatal care.

## Figures and Tables

**Table 1:** Baseline differences by study condition.

	Centering Pregnancy (n = 216)	Non-Centering Pregnancy (n = 1159)	*p*
Covariates or Matching Variables			
BMI (1 = overweight or obese and 0 = underweight or normal weight)	90.82%	88.90%	0.413
Race (1 = Black and 0 = non-Black)	67.13%	33.13%	**0.000**
Ethnicity (1 = Hispanic/Latinx and 0 = non-Hispanic/Latinx)	13.43%	24.85%	**0.000**
Age	24.89	25.94	**0.006**
Marital status (1 = married and 0 = unmarried)	19.07%	31.06%	**0.000**
Insurance type (1 = privately insured and 0 = publicly insured)	27.83%	39.91%	**0.001**
Outcome variables			
Prenatal care visits attended	10.86	9.02	**0.000**
Gestational age in weeks	37.97	36.70	**0.000**
Preterm birth (1 = less than 37 weeks and 0 = equal to or greater than 37 weeks)	8.80%	23.55%	**0.000**
Perinatal death (1 = demise and 0 = living)	1.39%	4.34%	**0.039**
Delivery type (1 = vaginal delivery and 0 = C-section)	70.83%	74.78%	0.224
Infant birth weight (oz)	110.65	101.82	**0.000**
Low birth weight (less than 88.18 oz or not)	9.35%	20.78%	**0.000**
Very low birth weight (less than 52.91 oz or not)	3.74%	10.78%	**0.001**
Gestational weight gain (lbs)	27.76	23.96	**0.001**
Exclusive breastfeeding at discharge (yes or no)	96.76%	94.22%	0.129
Exclusive breastfeeding at 6-week postpartum visit (yes or no)	34.26%	38.83%	0.205

Note: Significant *p*-values (i.e., *p* < 0.05) are bolded for clarity.

**Table 2:** Estimation results by treatment effect models.

	n	Regression Adjustment (RA)			Augmented Inverse-Probability Weighting (AIPW)			Inverse-Probability-Weighted Regression Adjustment (IPWRA)			Nearest-Neighbor Matching (NNM)			Propensity Score Matching (PSM)		
		Coef.	Std. Err.	*p*	Coef.	Std. Err.	*p*	Coef.	Std. Err.	*p*	Coef.	Std. Err.	*p*	Coef.	Std. Err.	*p*
Prenatal care visits	1035	2.116	0.283	**0.000**	2.221	0.273	**0.000**	2.175	0.267	**0.000**	1.854	0.355	**0.000**	1.849	0.420	**0.000**
Gestational age	1269	1.229	0.265	**0.000**	1.321	0.253	**0.000**	1.310	0.247	**0.000**	1.334	0.297	**0.000**	1.266	0.326	**0.000**
Preterm birth (less than 37 weeks or not)	1269	−0.119	0.030	**0.000**	−0.135	0.028	**0.000**	−0.130	0.027	**0.000**	−0.135	0.033	**0.000**	−0.135	0.036	**0.000**
Perinatal death (dead or alive)	1266										−0.036	0.012	**0.002**	−0.037	0.008	**0.000**
Delivery type (vaginal or C-section)	1263	−0.042	0.040	0.294	−0.053	0.041	0.190	−0.053	0.039	0.177	−0.091	0.050	0.066	−0.015	0.053	0.775
Infant birth weight (oz)	1261	10.804	2.088	**0.000**	11.560	1.994	**0.000**	11.450	1.967	**0.000**	11.899	2.253	**0.000**	13.338	1.801	**0.000**
Low birth weight (less than 88.18 oz or not)	1261	−0.120	0.025	**0.000**	−0.129	0.023	**0.000**	−0.126	0.023	**0.000**	−0.141	0.026	**0.000**	−0.158	0.018	**0.000**
Very low birth weight (less than 52.91 oz or not)	1261	−0.064	0.018	**0.000**	−0.067	0.017	**0.000**	−0.066	0.017	**0.000**	−0.067	0.019	**0.000**	−0.080	0.013	**0.000**
Gestational weight gain (lbs)	1018	3.831	1.346	**0.004**	3.472	1.325	**0.009**	3.494	1.313	**0.008**	3.481	1.655	**0.035**	3.172	1.518	**0.037**
Exclusive breastfeeding at discharge (yes or no)	1272	0.023	0.015	0.119	0.025	0.014	0.085	0.025	0.014	0.085	0.010	0.021	0.643	0.022	0.018	0.215
Exclusive breastfeeding at 6-week postpartum visit (yes or no)	1272	−0.013	0.043	0.761	−0.033	0.043	0.447	−0.030	0.041	0.473	−0.027	0.049	0.580	−0.005	0.042	0.907

Note: Significant *p*-values (i.e., *p* < 0.05) are bolded for clarity. With regard to the variable perinatal death, treatment effect estimates for regression adjustment, augmented inverse-probability weighting, and inverse-probability-weighted regression adjustment did not converge; therefore, the estimation results could not be reported.

## Data Availability

The dataset analyzed in this study is not publicly available to protect the privacy and confidentiality of participants, in compliance with IRB regulations. However, the dataset may be available upon request, subject to approval by the Institutional Review Boards (IRBs).
